# Close phylogenetic relationship between Angolan and Romanian HIV-1 subtype F1 isolates

**DOI:** 10.1186/1742-4690-6-39

**Published:** 2009-04-22

**Authors:** Monick L Guimarães, Ana Carolina P Vicente, Koko Otsuki, Rosa Ferreira FC da Silva, Moises Francisco, Filomena Gomes da Silva, Ducelina Serrano, Mariza G Morgado, Gonzalo Bello

**Affiliations:** 1 Laboratório de AIDS & Imunologia Molecular, Instituto Oswaldo Cruz – FIOCRUZ, Rio de Janeiro, Brazil; 2 Laboratório de Genética Molecular de Microorganismos, Instituto Oswaldo Cruz – FIOCRUZ, Rio de Janeiro, Brazil; 3 Instituto Nacional de Saúde Publica de Angola – Ministério da Saúde, Luanda, Angola; 4 Instituto Nacional de Luta Contra SIDA – Ministério da Saúde, Luanda, Angola

## Abstract

**Background:**

Here, we investigated the phylogenetic relationships of the HIV-1 subtype F1 circulating in Angola with subtype F1 strains sampled worldwide and reconstructed the evolutionary history of this subtype in Central Africa.

**Methods:**

Forty-six HIV-1-positive samples were collected in Angola in 2006 and subtyped at the *env*-gp41 region. Partial *env*-gp120 and *pol-RT *sequences and near full-length genomes from those *env*-gp41 subtype F1 samples were further generated. Phylogenetic analyses of partial and full-length subtype F1 strains isolated worldwide were carried out. The onset date of the subtype F1 epidemic in Central Africa was estimated using a Bayesian Markov chain Monte Carlo approach.

**Results:**

Nine Angolan samples were classified as subtype F1 based on the analysis of the *env*-gp41 region. All nine Angolan sequences were also classified as subtype F1 in both *env-gp120 *and *pol-RT *genomic regions, and near full-length genome analysis of four of these samples confirmed their classification as "pure" subtype F1. Phylogenetic analyses of subtype F1 strains isolated worldwide revealed that isolates from the Democratic Republic of Congo (DRC) were the earliest branching lineages within the subtype F1 phylogeny. Most strains from Angola segregated in a monophyletic group together with Romanian sequences; whereas South American F1 sequences emerged as an independent cluster. The origin of the subtype F1 epidemic in Central African was estimated at 1958 (1934–1971).

**Conclusion:**

"Pure" subtype F1 strains are common in Angola and seem to be the result of a single founder event. Subtype F1 sequences from Angola are closely related to those described in Romania, and only distantly related to the subtype F1 lineage circulating in South America. Original diversification of subtype F1 probably occurred within the DRC around the late 1950s.

## Background

Human immunodeficiency virus type 1 (HIV-1) sequences of the major group M are classified into nine subtypes (A-D, F-H, J, and K), six sub-subtypes (A1–A4, and F1–F2), and a variety of circulating recombinant forms (CRFs) and unique recombinant forms (URFs) (Los Alamos HIV sequence database: ). The subtype F1 causes a small number of infections globally (<1%) [1]; but it is particularly prevalent in some specific countries from Europe, South America, and Central Africa, either in its non-recombinant form, or as part of recombinant genomes.

In Europe, non-recombinant subtype F1 strains reach a high prevalence (>70%) among Romanian children and adults [2-5]. This Romanian epidemic was probably caused by the introduction of one subtype F1 virus into the adult population sometime before it appeared in 1989 among institutionalized children [6]. In South America, subtype F1 and mainly BF1 recombinant variants are prevalent (>10%) in countries from the Southern cone (Argentina, Brazil, Chile, Paraguay, and Uruguay), particularly among intravenous drug users and heterosexual populations [7-22]. A previous study suggested that the subtype F1 and BF1 epidemics in South America were initiated by the introduction, through Brazil, of a single founder subtype F1 strain around the middle-late 1970s [23]. Several studies have shown that the South American and Romanian epidemics are the result of distinct subtype F1 introductions [3,4,6,23]; but the geographic epicenter(s) of these subtype F1 epidemics is one of the most puzzling aspects in the worldwide spread of the HIV-1.

The overall prevalence of subtype F1 in Africa is very low, and most of the subtype F1 infections initially described in the continent were from the Democratic Republic of Congo (DRC). Although subtype F1 forms represent a small percentage (<5%) of the HIV-1 strains circulating in the DRC [24-30], sporadic cases of subtype F1 and CRF05_DF infections have been reported in Belgium and the Netherlands among individuals with a direct epidemiological link to the DRC [31-34]; this indicates that this country could be an important epicenter of the world-wide dispersion of both pure and recombinant subtype F1 strains. The most extensive phylogenetic analysis of subtype F1 strains circulating worldwide performed to date, however, revealed that although sequences from the DRC fell in a basal position within the subtype F1 phylogeny, they were only weakly associated with the South American and Romanian clades [23].

Angola is a Central African country bordered by the DRC, Republic of the Congo, Namibia and Zambia. Like in the DRC, the HIV-1 epidemic in Angola is characterized by the circulation of all group M subtypes and sub-subtypes, a high number of URFs, and several unclassifiable sequences [35-37]. Two recent studies, based on the analysis of partial genome regions, described an unusual high prevalence of subtype F1 infections in Angola, ranging from 8% to 16% [35,37]. Angola maintains strong social, cultural and economic relationships with Brazil. Noteworthy, the estimated onset date of the subtype F1 epidemic in Brazil (and South America) was around 1975–1980 [23,38,39], coinciding with the beginning of the Angolan civil war in 1975 that was followed by a wave of emigration. These observations have lead us to suggest that the subtype F1 found in South America could have been originated from Angola [23].

To further test this hypothesis, we recovered nine partial and four full-length genome sequences from subtype F1 HIV-1 isolates from Angola and investigated the phylogenetic relationship of these strains with other subtype F1 strains isolated worldwide. We also estimated the onset year of the HIV-1 subtype F1 epidemic in Central Africa based on the analysis of 47 non-contemporary *env *gene sequences of African origin sampled over a period of 22 years (1984–2006).

## Results

### Identification of "pure" HIV-1 subtype F1 viruses in Angola

**Figure 1:**
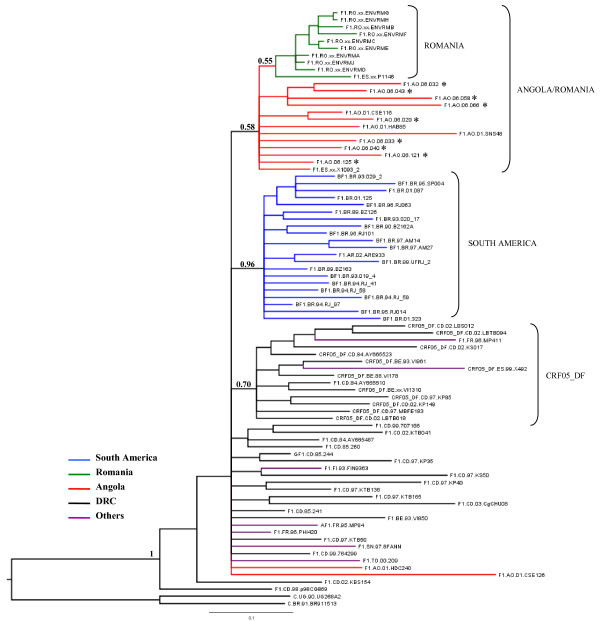
**Majority-rule Bayesian consensus tree of HIV-1 subtype F1 *env-gp120 *(310 bp) sequences**. Posterior probabilities are shown for key nodes. The names of HIV-1 isolates include reference to subtype, country of isolation, and year of isolation. The color of each branch within the subtype F1 cluster represents the country (or geographic region) of origin of sequence corresponding to that branch, according to the legend in the figure. The asterisks point at the subtype F1 Angolan sequences described in the present work. Brackets indicate the different monophyletic clusters identified. The trees were rooted using subtype C reference sequences as outgroups. Horizontal branch lengths are drawn to scale with the bar at the bottom indicating 0.1 nucleotide substitutions per site.

**Figure 2:**
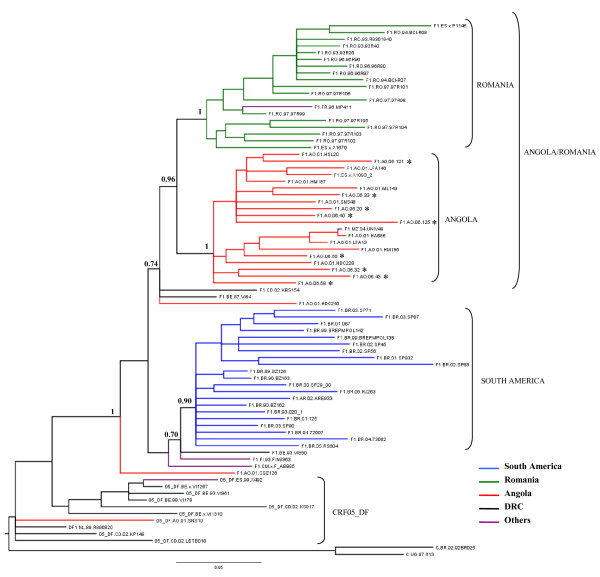
**Majority-rule Bayesian consensus tree of HIV-1 subtype F1 *pol-rt *(866 nt) sequences**. See legend of Fig. 1. Horizontal branch lengths are drawn to scale with the bar at the bottom indicating 0.05 nucleotide substitutions per site.

Among 46 samples collected in Angola in 2006, nine (20%) were classified as subtype F1 based on the analysis of the *env*-gp41 region (data not shown). These samples were further analyzed in the *env*-gp120 and *pol*-rt regions, and all nine Angolan sequences confirmed their classification as subtype F1 in both genomic regions (Figs. 1 and 2). To confirm the circulation of non-recombinant subtype F1 strains in Angola, we expanded the genetic characterization of four strains to near-full length genome sequencing. According to the REGA HIV-1 Subtyping Tool and bootscanning analyses, no evidence of inter-subtype recombination was found among the four samples analyzed, confirming their classification as "pure" subtype F1 strains. Twelve "pure" subtype F1 strains were described in the literature to date (Table 1). Six were isolated in South America, five from Brazil [15,40,41], and one from Argentina [23]. The other six strains have a reported epidemiological link to the DRC, Kenya, Chad, Romania and Angola, but were isolated in Belgium [33], Finland [33], France [42], and Spain (Sierra *et al*, unpublished results) (Table 1). Thus, the four new subtype F1 Angolan sequences described in the present study are the first "pure" HIV-1 subtype F1 strains isolated in Central Africa up to date.

**Table 1:** HIV-1 subtype F1 data sets.

Genome region	Geographic origin	Country of Isolation	F1 (New*)	CRF05_DF
*env*	Angola	Angola	14 (9)	-
		
		Spain	1	-
	
	DRC	DRC	17	8
		
		Belgium	1	3
	
	Africa	Senegal/Chad	2	-
		
		Finland/France/Spain	4	1
	
	South America	Brazil	20	-
		
		Argentina	1	-
	
	Romania	Romania	9	-
		
		Spain	1	-

*pol*	Angola	Angola	20 (9)	1
		
		Spain	1	-
	
	DRC	DRC	1	3
		
		Belgium/Netherlands	2	5
	
	Africa	Cameroon/Mozambique	2	-
		
		Finland/France/Spain	2	1
	
	South America	Brazil	20	-
		
		Argentina	1	-
	
	Romania	Romania	16	-
		
		Spain	2	-

Complete Genome	Angola	Angola	4 (4)	-
		
		Spain	1	-
	
	DRC	Belgium	1	-
	
	Africa	Finland	1	-
		
		France	1	-
	
	South America	Brazil	5	-
		
		Argentina	1	-
	
	Romania	Spain	2	-

* Described in the present work.

### Phylogenetic analysis of env, pol, and full-length subtype F1 sequences

To investigate the phylogenetic relationship among HIV-1 subtype F1 isolated in Angola and other subtype F1 strains sampled worldwide, the new subtype F1 *env-gp120 *(310-bp) Angolan sequences and six subtype F1 *env-gp120 *Angolan sequences described previously were aligned with all strains from African and Romanian origin and a subset of strains of South American origin that were subtype F1 in the genome fragment analyzed. These subset strain sequences are available at the Los Alamos HIV database. This approach resulted in a final data set of 82 subtype F1 *env *sequences (Angola = 15, DRC = 29, Romania = 10, South America = 21, others = 7) (Table 1). The resulting Bayesian phylogenetic tree (Fig. 1) showed that isolates from the DRC occupy the most basal positions in the subtype F1 phylogeny, confirming their older radiation and indicating that the original diversification of subtype F1 probably occurred within or near the DRC. Two isolates from Angola previously described (AO.01.CSE126 and AO.01.HDC240), and isolates from other African countries also fell at the base of the tree, intermixed among strains from the DRC. Most subtype F1 sequences from Angola, however, segregated in a monophyletic group nested among the DRC strains, together with subtype F1 sequences from Romania. Within this Angola/Romania clade, isolates from Angola occupied basal positions whereas Romanian isolates branched as a monophyletic sub-cluster. The South American sequences, and the subtype F1 *env *segments derived from CRF05_DF strains formed two additional (and independent) monophyletic clusters nested within the basal strains from the DRC. The support of these distinct monophyletic clusters was generally low (posterior probability, *PP *< 70%), with the exception of the South American monophyletic group (*PP *= 96%).

To confirm this tree topology, a phylogenetic analysis of the larger *pol*-*RT *gene fragment (866-bp) was performed. The new subtype F1 *pol-RT *Angolan sequences and 12 subtype F1 *pol-RT *Angolan sequences previously described, were aligned with all strains from African and Romanian origin and a subset of strains of South American origin, available at the Los Alamos HIV database, that were subtype F1 in the genome fragment analyzed. The final dataset contained a total of 67 subtype F1 *pol *sequences (Angola = 21, DRC = 3, Romania = 18, South America = 21, others = 4) (Table 1). Ten F1/D recombinant *pol *sequences from CRF05_DF strains were also included. The overall topology of the resulting *pol *Bayesian tree was similar to the *env *tree (Fig. 2). This analysis showed the clustering of the Angolan and Romanian strains in a highly supported monophyletic group (*PP *= 94%). Within this monophyletic group, strains from Angola and Romania segregated into two separate sub-groups (PP = 100%). One isolate from France (FR.96.MP411), and another one from Mozambique (MZ.04.UNV49) also segregated within the Romanian and Angolan sub-clusters, respectively. South American strains formed an independent monophyletic lineage (*PP *= 91%) that was only weakly associated with two reference strains isolated in Belgium (BE.93.VI850) and Finland (FI.93.FIN9363). The three strains from the DRC occupied the most ancestral positions within subtype F1, together with the Angolan strains AO.01.CSE126 and AO.01.HDC240, consistent with the *env *tree. As expected, the F1/D recombinant *pol *fragments of the CRF05_DF strains appeared outside the subtype F1 group.

**Figure 3:**
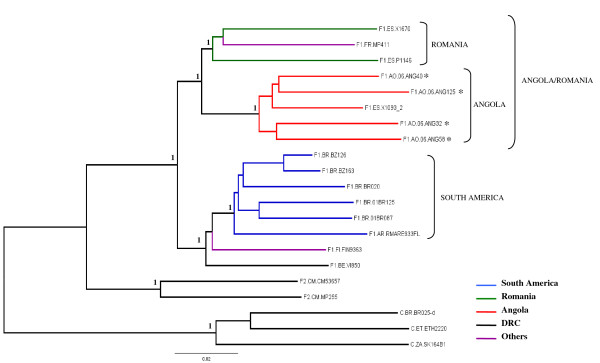
**Majority-rule Bayesian consensus tree of HIV-1 subtype F1 near full-length (~8.5 kb) strains**. Posterior probabilities are shown for key nodes. The names of HIV-1 isolates include reference to subtype and country of isolation. The color of each branch within the subtype F1 cluster represents the country (or geographic region) of origin of sequence corresponding to that branch, according to the legend in the figure. The asterisks points at the subtype F1 Angolan sequences described in the present work. Brackets mark the different subtype F1 monophyletic clusters identified. Subtypes F2 and C reference sequences were used as outgroup. Horizontal branch lengths are drawn to scale with the bar at the bottom indicating 0.02 nucleotide substitutions per site.

Finally, the evolutionary relationship among all 16 "pure" subtype F1 strains was investigated. Phylogenetic analysis of near FL (~8.5-kb) subtype F1 strains showed that Angolan and Romanian sequences segregated into two separate but highly related clusters, consistent with the *pol *tree topology; whereas South American strains form an independent monophyletic lineage (Fig. 3). The reference strain isolated in France (FR.96.MP411) segregated with the Romanian sequences, while the two strains isolated in Belgium (BE.93.VI850) and Finland (FI.93.FIN9363) clustered with the South American monophyletic lineage (Fig. 3). Maximum Likelihood analyses yielded *env*, *pol*, and FL trees with basically the same topology (data not shown). These analyses confirmed that subtype F1 strains from Angola and Romania are more related to each other than to any other subtype F1 strains isolated elsewhere.

### Timing the origin of the HIV-1 subtype F1 in Central Africa

To estimate the date of the root of the Central African subtype F1, a total of 49 subtype-F1 *env *(310-bp) sequences from African origin (*Angola *= 14; *DRC *= 28; *others *= 7) sampled from 1984 to 2006 were used; including 11 subtype-F1 *env *sequences derived from CRF05_DF strains. Evolutionary parameters were estimated under strict and relaxed clock models as implemented in BEAST v1.7. The coefficient of variation under the relaxed clock model was 0.24 (95% Highest Posterior Density [HPD]: 0.12–0.36); indicating detectable variation in evolutionary rates among branches. Despite this, the median evolutionary rate estimated for this data set under both strict (3.3 × 10^-3 ^[95% HPD: 2.2 × 10^-3^-4.5 × 10^-3^] *subst./site/year*) and relaxed (3.2 × 10^-3 ^[95% HPD: 1.9 × 10^-3^-4.5 × 10^-3^] *subst./site/year*) clock models was very close. According to this *env *dataset the time of the most recent common ancestor(*T*_mrca_) of the subtype F1 epidemic was estimated to be around 1955 (95% HPD: 1932–1970; strict clock) and 1958 (95% HPD: 1934–1971; relaxed clock).

## Discussion

Two previous studies described a high prevalence (8–16%) of HIV-1 subtype F1 infections in Angola, based on the analysis of partial genomic regions [35,37]. In the present work, nine (20%) out of 46 HIV-1 samples recovered in Angola in 2006 were classified as subtype F1 after analysis of the *env*-gp41 region, confirming the previous observations. A considerable proportion (~40%) of *env *subtype F1 viruses originating in the DRC display a F1/D recombinant profile in *pol *and are, in fact, CRF05_DF strains. All nine *env *subtype F1 Angolan sequences identified in our study, however, exhibited a non-recombinant subtype F1 profile at *pol*. Four samples were further subjected to near full-length genome sequencing and were classified as non-recombinant F1 strains, showing for the first time that "pure" HIV-1 subtype F1 viruses are common in Angola. The detection of "pure" subtype F1 genomes at relatively high prevalence in Angola is surprising considering the co-circulation of all group M subtypes and the great number of URFs described in the country [35-37].

Phylogenetic analyses of subtype F1 strains isolated worldwide suggest that the original diversification of subtype F1 occurred within the DRC and subsequently spread to Angola, Romania and South America. Despite the intense population mobility between Angola and the neighbor DRC, subtype F1 variants from both countries were not highly intermixed in the phylogenetic trees. Most Angolan subtype F1 strains segregated in a monophyletic cluster nested within the strains from the DRC; suggesting that most subtype F1 infections in Angola derived from a single founder event. Only two Angolan samples described previously branched outside this major Angolan cluster, and probably represent independent introductions of subtype F1 into Angola. Thus, the subtype F1 epidemic in Angola seems to be mainly driven by internal spread of a local subtype F1 variant introduced sometime in the past, rather than by repeated introductions of subtype F1 variants from neighboring countries.

The high prevalence of subtype F1 in Angola, the strong relationships between Angola and Brazil, and the coincidence between the estimated onset date of the subtype F1 epidemic in Brazil (around 1975–1980) [23,38,39] and the beginning of the Angolan civil war (in 1975) lead us to suggest that the subtype F1 found in Brazil (and South America) probably originates from Angola. All phylogenetic analyses conducted in sub-genomic and full-genome regions showed, however, that the South American and Angolan F1 sequences shared a distant common ancestor and segregated in independent clusters within the subtype F1 phylogeny; suggesting no direct epidemiological link between these lineages. Two strains isolated in Belgium (BE.93.VI850) and Finland (FI.93.FIN9363), but probably originating in the DRC and Kenya, were the African sequences most closely related to the South American cluster. Whether the South American subtype F1 lineage originated from the DRC or from some lineage circulating in Angola as a minor form is unclear.

Surprisingly, all phylogenetic analyses conducted in this study revealed that the subtype F1 variants from Angola and Romania are more related to each other than to any other subtype F1 strains isolated elsewhere. Analysis of *env *region reveals that isolates from Angola occupied basal positions within the Angola/Romania clade, whereas Romanian isolates branched as a monophyletic sub-cluster, indicating that Angola could be the epicenter of the subtype F1 epidemic that spread into Romania. Analyses of *pol *and full-length genome, however, showed that Romanian and Angolan subtype F1 sequences segregate in two highly related but distinct sub-clusters, suggesting that both epidemics could have arisen independently from closely related subtype F1 strains probably introduced from the DRC.

The median *T*_mrca _of the African subtype F1 epidemic was estimated to be around the late 1950s, more than 30 years later than the estimated emergence of the HIV-1 group M in Central Africa [43,44]. Previous studies also place the date of origin of the subtype C epidemic in Africa between the middle 1950s and the middle 1960s [45-47], supporting a similar emergence date of the two subtype epidemics. The simultaneous appearance of group M subtypes suggests that some extrinsic factors could have played roles in producing and spreading high numbers of HIV-1 infections in the DRC during the period of 1950–1960. Of note, this period coincides with massive people movements from rural areas to the major cities (e.g., the population of Kinshasa increased almost 10-fold from 1940 to 1960) [48], and dramatic increases in the number of unsterile injections [49] in Central Africa. In such new high-risk social networks, some of the pre-existing HIV-1 group M lineages may have more opportunity for rapid local expansion, and subsequent dissemination out of the epicentre resulting in the global subtypes.

## Conclusion

The subtype F1 epidemic, as with most HIV-1 group M clades, probably emerged in the DRC around the late 1950s and subsequently spread locally and globally. Our results suggest that non-recombinant subtype F1 HIV-1 strains are present in Angola at a prevalence higher than in any other African country, and this could have important implications for vaccine design. The subtype F1 epidemic in Angola has its own characteristics, different from the neighbouring DRC; and it is highly related to the subtype F1 epidemic spreading in Romania. The exact origin of the Romanian and South American subtype F1 epidemics remains unclear; and a denser sampling of subtype F1 sequences from Central Africa will be needed in order to reconstruct the history of these epidemics.

## Methods

### Study population

Forty-six HIV-1-positive samples were collected in 2006 at the "Hospital Esperança" in Luanda, Angola, with the approval of local Ethical Committee. Nine samples were classified as subtype F1 based on the analysis of the envelope (*env*)-gp41 region, and further subjected to the analysis of the *env-gp120 *and the polymerase-reverse transcriptase (*pol*-rt) regions. Four samples were subjected to near full-length (FL) genome analysis. Additionally, five *env-gp120 *and 11 *pol*-rt subtype F1 Angolan sequences described previously [36,37], and one FL subtype F1 sequence of Angolan origin isolated in Spain (Sierra *et al*, unpublished results) were also downloaded from the Los Alamos HIV Sequence database.

### Amplification and sequencing of HIV-1 DNA

DNA samples were extracted from 200 μl of whole blood using a QIAamp DNA kit (Qiagen Inc., CA, U.S.A.), according to the manufacturer's protocol. Partial *env *and *pol *regions were PCR-amplified using nested primers as previously described [50]. Amplification of near-FL HIV-1 genomes (~8.5-kb) was obtained by nested PCR of four overlapping fragments of 2,100-bp to 3,100-bp each. Primer sequences and PCR conditions used for nested amplifications are available upon request. PCR products were purified using the Qiagen PCR purification kit (Qiagen) according to the manufacturer's protocol. Purified DNA was sequenced by using the ABI BigDye Terminator v.3.1 cycle sequencing ready reaction kit (Applied Biosystem, CA, U.S.A), and processed with an automated ABI 3100 Genetic Analyzer (Applied Biosystem). Sequence electropherograms were edited and assembled with the Seqman v.7.0 program (DNASTAR).

### Sequence alignments

Nucleotide sequences were aligned using CLUSTAL X program [51] and later hand edited. All positions with alignment gaps and regions of ambiguous alignment were removed. Three distinct alignments were used to investigate the phylogenetic relationship among subtype F1 sequences isolated worldwide (Table 1). The first alignment of 310-bp spanned the V3 region of the *env *gene (positions 7050 to 7374 relative to HXB2) and included a total of 82 subtype F1 sequences isolated worldwide. The second alignment of 866-bp covered part of the *pol *(reverse transcriptase) gene (positions 2550–3415 relative to HXB2) and contained 67 subtype F1 sequences from all over the world, and 10 F1/D recombinant sequences derived from CRF05_DF strains. The third alignment of ~8,400-bp (positions 790 to 9084 relative to HXB2 reference strain) contained 16 subtype F1 FL sequences (four new and 12 available in the Los Alamos HIV database). A fourth alignment of 310-bp, spanning the same region of the *env *gene previously described, was used to estimate the onset date of the subtype F1 epidemic in Central Africa and contained 49 subtype F1 sequences of African origin with a known sampling year. All alignments are available from the authors upon request.

### HIV-1 subtype classification

Analyses of HIV-1 subtypes and recombination were performed using: 1) the REGA HIV-1 Subtyping Tool [52]; and 2) bootscanning analysis (sliding window of 400 bp, incremental steps of 10 bases, and the Kimura two-parameter model) as implemented in Simplot version 2.5 [53]. Bootstrap support was calculated based on 100 re-samplings.

### Phylogenetic analyses

The best-fit model of nucleotide substitution was selected using Modeltest [54] resulting in the general time-reversible nucleotide substitution model with gamma-distributed rate heterogeneity among sites and a proportion of invariable sites (GTR+I+Γ) in all cases. Phylogenetic tree reconstructions were performed by Bayesian method using MrBayes version 3.1.2 [55]. For each dataset, two runs of four chains each (one cold and three heated, temp = 0.20) were run for 50 × 10^6 ^generations, with a burn-in of 10 × 10^6 ^generations. Convergence of parameters was assessed by calculating the Effective Sample Size (ESS) using TRACER v1.4 [56], excluding an initial 10% for each run. All parameter estimates for each run showed ESS values >100. A final Bayesian majority-rule consensus tree was obtained for each data set. Maximum Likelihood trees were reconstructed with PhyML [57] using an online web server [58] and assessing phylogenetic confidence by bootstrap with 100 replicates. Trees were visualized using the FigTree v1.1.2 program available at .

### Estimation of evolutionary rates and dates

Estimates of the evolutionary rate (*μ*, units are nucleotide substitutions per site per year, *subst./site/year*) and the time of the most recent common ancestor (*T*_mrca_, years) of the Central African subtype F1 epidemic were performed using a Bayesian Markov Chain Monte Carlo (MCMC) approach as implemented in BEAST v1.7 [59,60]. The time span covered by the African subtype F1 *env *sequences (i.e., 22 years) was sufficient to reliably estimate the evolutionary parameters under a chronological time-scale employing the dates of the sequences. Analyses were performed with a Bayesian Skyline coalescent tree prior [61], under the GTR+I+Γ nucleotide substitution model, and using both a strict and a relaxed (uncorrelated Lognormal model) molecular clock [62]. Three separate MCMC chains were run for 1 × 10^7 ^generations with a burn-in of 1 × 10^6^. BEAST output was analyzed using TRACER v1.4, with uncertainty in parameter estimates reflected in the 95% Highest Posterior Density (HPD) intervals. All Bayesian MCMC independent runs converged to almost identical values for all parameters, and the ESS values for parameter estimates were >100. The results reported are the combined estimates of the three independent runs.

### GenBank accession numbers

Sequences were deposited in GenBank under accession numbers FJ900256 to FJ900269.

## Competing interests

The authors declare that they have no competing interests.

## Authors' contributions

GB, MLG, and MM conceived and designed the study. RFCFS, MF, FGS, and DS were responsible for patients' recruitment and sample collection in Angola. RFCFS, KO, and ACPV performed the initial characterization of samples in the *env*-gp41 region, and subsequent analysis of the *env*-gp120 region. MLG conducted the characterization of the *pol *region and the full-length genome analysis. GB performed the phylogenetic and coalescent analyses. GB wrote the first draft and all authors contributed to the final version of the paper.

## References

[B1] Hemelaar J, Gouws E, Ghys PD, Osmanov S (2006). Global and regional distribution of HIV-1 genetic subtypes and recombinants in 2004. Aids.

[B2] Dumitrescu O, Kalish ML, Kliks SC, Bandea CI, Levy JA (1994). Characterization of human immunodeficiency virus type 1 isolates from children in Romania: identification of a new envelope subtype. J Infect Dis.

[B3] Bandea CI, Ramos A, Pieniazek D, Pascu R, Tanuri A, Schochetman G, Rayfield MA (1995). Epidemiologic and evolutionary relationships between Romanian and Brazilian HIV-subtype F strains. Emerg Infect Dis.

[B4] Apetrei C, Loussert-Ajaka I, Collin G, Letourneur F, Duca M, Saragosti S, Simon F, Brun-Vezinet F (1997). HIV type 1 subtype F sequences in Romanian children and adults. AIDS Res Hum Retroviruses.

[B5] Apetrei C, Necula A, Holm-Hansen C, Loussert-Ajaka I, Pandrea I, Cozmei C, Streinu-Cercel A, Pascu FR, Negut E, Molnar G, Duca M, Pecec M, Brun-Vezinet F, Simon F (1998). HIV-1 diversity in Romania. Aids.

[B6] Op De Coul E, Burg R van den, Asjo B, Goudsmit J, Cupsa A, Pascu R, Usein C, Cornelissen M (2000). Genetic evidence of multiple transmissions of HIV type 1 subtype F within Romania from adult blood donors to children. AIDS Res Hum Retroviruses.

[B7] Sabino EC, Diaz RS, Brigido LF, Learn GH, Mullins JI, Reingold AL, Duarte AJ, Mayer A, Busch MP (1996). Distribution of HIV-1 subtypes seen in an AIDS clinic in Sao Paulo City, Brazil. Aids.

[B8] Morgado MG, Guimaraes ML, Gripp CB, Costa CI, Neves I, Veloso VG, Linhares-Carvalho MI, Castello-Branco LR, Bastos FI, Kuiken C, Castilho EA, Galvao-Castro B, Bongertz V (1998). Molecular epidemiology of HIV-1 in Brazil: high prevalence of HIV-1 subtype B and identification of an HIV-1 subtype D infection in the city of Rio de Janeiro, Brazil. Evandro Chagas Hospital AIDS Clinical Research Group. J Acquir Immune Defic Syndr Hum Retrovirol.

[B9] Bongertz V, Bou-Habib DC, Brigido LF, Caseiro M, Chequer PJ, Couto-Fernandez JC, Ferreira PC, Galvao-Castro B, Greco D, Guimaraes ML, Linhares de Carvalho MI, Morgado MG, Oliveira CA, Osmanov S, Ramos CA, Rossini M, Sabino E, Tanuri A, Ueda M (2000). HIV-1 diversity in Brazil: genetic, biologic, and immunologic characterization of HIV-1 strains in three potential HIV vaccine evaluation sites. Brazilian Network for HIV Isolation and Characterization. J Acquir Immune Defic Syndr.

[B10] Masciotra S, Livellara B, Belloso W, Clara L, Tanuri A, Ramos AC, Baggs J, Lal R, Pieniazek D (2000). Evidence of a high frequency of HIV-1 subtype F infections in a heterosexual population in Buenos Aires, Argentina. AIDS Res Hum Retroviruses.

[B11] Thomson MM, Villahermosa ML, Vazquez-de-Parga E, Cuevas MT, Delgado E, Manjon N, Medrano L, Perez-Alvarez L, Contreras G, Carrillo MG, Salomon H, Najera R (2000). Widespread circulation of a B/F intersubtype recombinant form among HIV-1-infected individuals in Buenos Aires, Argentina. Aids.

[B12] Russell KL, Carcamo C, Watts DM, Sanchez J, Gotuzzo E, Euler A, Blanco JC, Galeano A, Alava A, Mullins JI, Holmes KK, Carr JK (2000). Emerging genetic diversity of HIV-1 in South America. Aids.

[B13] Guimaraes ML, Bastos FI, Telles PR, Galvao-Castro B, Diaz RS, Bongertz V, Morgado MG (2001). Retrovirus infections in a sample of injecting drug users in Rio de Janeiro City, Brazil: prevalence of HIV-1 subtypes, and co-infection with HTLV-I/II. J Clin Virol.

[B14] Avila MM, Pando MA, Carrion G, Peralta LM, Salomon H, Carrillo MG, Sanchez J, Maulen S, Hierholzer J, Marinello M, Negrete M, Russell KL, Carr JK (2002). Two HIV-1 epidemics in Argentina: different genetic subtypes associated with different risk groups. J Acquir Immune Defic Syndr.

[B15] Hierholzer J, Montano S, Hoelscher M, Negrete M, Hierholzer M, Avila MM, Carrillo MG, Russi JC, Vinoles J, Alava A, Acosta ME, Gianella A, Andrade R, Sanchez JL, Carrion G, Russell K, Robb M, Birx D, McCutchan F, Carr JK (2002). Molecular Epidemiology of HIV Type 1 in Ecuador, Peru, Bolivia, Uruguay, and Argentina. AIDS Res Hum Retroviruses.

[B16] Brindeiro RM, Diaz RS, Sabino EC, Morgado MG, Pires IL, Brigido L, Dantas MC, Barreira D, Teixeira PR, Tanuri A (2003). Brazilian Network for HIV Drug Resistance Surveillance (HIV-BResNet): a survey of chronically infected individuals. Aids.

[B17] Espinosa A, Vignoles M, Carrillo MG, Sheppard H, Donovan R, Peralta LM, Rossi D, Radulich G, Salomon H, Weissenbacher M (2004). Intersubtype BF recombinants of HIV-1 in a population of injecting drug users in Argentina. J Acquir Immune Defic Syndr.

[B18] Quarleri JF, Rubio A, Carobene M, Turk G, Vignoles M, Harrigan RP, Montaner JS, Salomon H, Gomez-Carrillo M (2004). HIV type 1 BF recombinant strains exhibit different pol gene mosaic patterns: descriptive analysis from 284 patients under treatment failure. AIDS Res Hum Retroviruses.

[B19] Teixeira SL, Bastos FI, Telles PR, Hacker MA, Brigido LF, de FOCA, Bongertz V, Morgado MG (2004). HIV-1 infection among injection and ex-injection drug users from Rio de Janeiro, Brazil: prevalence, estimated incidence and genetic diversity. J Clin Virol.

[B20] Montano SM, Sanchez JL, Laguna-Torres A, Cuchi P, Avila MM, Weissenbacher M, Serra M, Vinoles J, Russi JC, Aguayo N, Galeano AH, Gianella A, Andrade R, Arredondo A, Ramirez E, Acosta ME, Alava A, Montoya O, Guevara A, Manrique H, Lama JR, de la Hoz F, Sanchez GI, Ayala C, Pacheco ME, Carrion G, Chauca G, Perez JJ, Negrete M, Russell KL, Bautista CT, Olson JG, Watts DM, Birx DL, Carr JK (2005). Prevalences, genotypes, and risk factors for HIV transmission in South America. J Acquir Immune Defic Syndr.

[B21] Gomez-Carrillo M, Pampuro S, Duran A, Losso M, Harris DR, Read JS, Duarte G, De Souza R, Soto-Ramirez L, Salomon H (2006). Analysis of HIV type 1 diversity in pregnant women from four Latin American and Caribbean countries. AIDS Res Hum Retroviruses.

[B22] Rios M, Delgado E, Perez-Alvarez L, Fernandez J, Galvez P, de Parga EV, Yung V, Thomson MM, Najera R (2007). Antiretroviral drug resistance and phylogenetic diversity of HIV-1 in Chile. J Med Virol.

[B23] Aulicino PC, Bello G, Rocco C, Romero H, Mangano A, Morgado MG, Sen L (2007). Description of the First Full-Length HIV Type 1 Subtype F1 Strain in Argentina: Implications for the Origin and Dispersion of This Subtype in South America. AIDS Res Hum Retroviruses.

[B24] Triques K, Bourgeois A, Saragosti S, Vidal N, Mpoudi-Ngole E, Nzilambi N, Apetrei C, Ekwalanga M, Delaporte E, Peeters M (1999). High diversity of HIV-1 subtype F strains in Central Africa. Virology.

[B25] Mokili JL, Wade CM, Burns SM, Cutting WA, Bopopi JM, Green SD, Peutherer JF, Simmonds P (1999). Genetic heterogeneity of HIV type 1 subtypes in Kimpese, rural Democratic Republic of Congo. AIDS Res Hum Retroviruses.

[B26] Vidal N, Peeters M, Mulanga-Kabeya C, Nzilambi N, Robertson D, Ilunga W, Sema H, Tshimanga K, Bongo B, Delaporte E (2000). Unprecedented degree of human immunodeficiency virus type 1 (HIV-1) group M genetic diversity in the Democratic Republic of Congo suggests that the HIV-1 pandemic originated in Central Africa. J Virol.

[B27] Yang C, Dash B, Hanna SL, Frances HS, Nzilambi N, Colebunders RC, St Louis M, Quinn TC, Folks TM, Lal RB (2001). Predominance of HIV type 1 subtype G among commercial sex workers from Kinshasa, Democratic Republic of Congo. AIDS Res Hum Retroviruses.

[B28] Kalish ML, Robbins KE, Pieniazek D, Schaefer A, Nzilambi N, Quinn TC, St Louis ME, Youngpairoj AS, Phillips J, Jaffe HW, Folks TM (2004). Recombinant viruses and early global HIV-1 epidemic. Emerg Infect Dis.

[B29] Yang C, Li M, Mokili JL, Winter J, Lubaki NM, Mwandagalirwa KM, Kasali MJ, Losoma AJ, Quinn TC, Bollinger RC, Lal RB (2005). Genetic diversification and recombination of HIV type 1 group M in Kinshasa, Democratic Republic of Congo. AIDS Res Hum Retroviruses.

[B30] Vidal N, Mulanga C, Bazepeo SE, Mwamba JK, Tshimpaka JW, Kashi M, Mama N, Laurent C, Lepira F, Delaporte E, Peeters M (2005). Distribution of HIV-1 variants in the Democratic Republic of Congo suggests increase of subtype C in Kinshasa between 1997 and 2002. J Acquir Immune Defic Syndr.

[B31] Lukashov VV, Kuiken CL, Boer K, Goudsmit J (1996). HIV type 1 subtypes in The Netherlands circulating among women originating from AIDS-endemic regions. AIDS Res Hum Retroviruses.

[B32] Heyndrickx L, Janssens W, Coppens S, Vereecken K, Willems B, Fransen K, Colebunders R, Vandenbruaene M, Groen G van der (1998). HIV type 1 C2V3 env diversity among Belgian individuals. AIDS Res Hum Retroviruses.

[B33] Laukkanen T, Carr JK, Janssens W, Liitsola K, Gotte D, McCutchan FE, Op de Coul E, Cornelissen M, Heyndrickx L, Groen G van der, Salminen MO (2000). Virtually full-length subtype F and F/D recombinant HIV-1 from Africa and South America. Virology.

[B34] Op de Coul E, Schoot A van der, Goudsmit J, Burg R van den, Janssens W, Heyndrickx L, Groen G van der, Cornelissen M (2000). Independent introduction of transmissible F/D recombinant HIV-1 from Africa into Belgium and The Netherlands. Virology.

[B35] Abecasis A, Paraskevis D, Epalanga M, Fonseca M, Burity F, Bartolomeu J, Carvalho AP, Gomes P, Vandamme AM, Camacho R (2005). HIV-1 genetic variants circulation in the North of Angola. Infect Genet Evol.

[B36] Bartolo I, Epalanga M, Bartolomeu J, Fonseca M, Mendes A, Gama A, Taveira N (2005). High genetic diversity of human immunodeficiency virus type 1 in Angola. AIDS Res Hum Retroviruses.

[B37] Bartolo I, Rocha C, Bartolomeu J, Gama A, Marcelino R, Fonseca M, Mendes A, Epalanga M, Silva PC, Taveira N (2008). Highly divergent subtypes and new recombinant forms prevail in the HIV/AIDS epidemic in Angola: New insights into the origins of the AIDS pandemic. Infect Genet Evol.

[B38] Bello G, Guimaraes ML, Morgado MG (2006). Evolutionary history of HIV-1 subtype B and F infections in Brazil. AIDS.

[B39] Bello G, Eyer-Silva WA, Couto-Fernandez JC, Guimaraes ML, Chequer-Fernandez SL, Teixeira SL, Morgado MG (2007). Demographic history of HIV-1 subtypes B and F in Brazil. Infect Genet Evol.

[B40] Gao F, Robertson DL, Carruthers CD, Morrison SG, Jian B, Chen Y, Barre-Sinoussi F, Girard M, Srinivasan A, Abimiku AG, Shaw GM, Sharp PM, Hahn BH (1998). A comprehensive panel of near-full-length clones and reference sequences for non-subtype B isolates of human immunodeficiency virus type 1. J Virol.

[B41] Sanabani S, Neto WK, Kalmar EM, Diaz RS, Janini LM, Sabino EC (2006). Analysis of the near full length genomes of HIV-1 subtypes B, F and BF recombinant from a cohort of 14 patients in Sao Paulo, Brazil. Infect Genet Evol.

[B42] Triques K, Bourgeois A, Vidal N, Mpoudi-Ngole E, Mulanga-Kabeya C, Nzilambi N, Torimiro N, Saman E, Delaporte E, Peeters M (2000). Near-full-length genome sequencing of divergent African HIV type 1 subtype F viruses leads to the identification of a new HIV type 1 subtype designated K. AIDS Res Hum Retroviruses.

[B43] Korber B, Muldoon M, Theiler J, Gao F, Gupta R, Lapedes A, Hahn BH, Wolinsky S, Bhattacharya T (2000). Timing the ancestor of the HIV-1 pandemic strains. Science.

[B44] Worobey M, Gemmel M, Teuwen DE, Haselkorn T, Kunstman K, Bunce M, Muyembe JJ, Kabongo JM, Kalengayi RM, Van Marck E, Gilbert MT, Wolinsky SM (2008). Direct evidence of extensive diversity of HIV-1 in Kinshasa by 1960. Nature.

[B45] Travers SA, Clewley JP, Glynn JR, Fine PE, Crampin AC, Sibande F, Mulawa D, McInerney JO, McCormack GP (2004). Timing and reconstruction of the most recent common ancestor of the subtype C clade of human immunodeficiency virus type 1. J Virol.

[B46] Rousseau CM, Learn GH, Bhattacharya T, Nickle DC, Heckerman D, Chetty S, Brander C, Goulder PJ, Walker BD, Kiepiela P, Korber BT, Mullins JI (2007). Extensive intrasubtype recombination in South African human immunodeficiency virus type 1 subtype C infections. J Virol.

[B47] Walker PR, Pybus OG, Rambaut A, Holmes EC (2005). Comparative population dynamics of HIV-1 subtypes B and C: subtype-specific differences in patterns of epidemic growth. Infect Genet Evol.

[B48] Chitnis A, Rawls D, Moore J (2000). Origin of HIV type 1 in colonial French Equatorial Africa?. AIDS Res Hum Retroviruses.

[B49] Marx PA, Alcabes PG, Drucker E (2001). Serial human passage of simian immunodeficiency virus by unsterile injections and the emergence of epidemic human immunodeficiency virus in Africa. Philos Trans R Soc Lond B Biol Sci.

[B50] Bello G, Passaes CP, Guimaraes ML, Lorete RS, Matos Almeida SE, Medeiros RM, Alencastro PR, Morgado MG (2008). Origin and evolutionary history of HIV-1 subtype C in Brazil. Aids.

[B51] Thompson JD, Gibson TJ, Plewniak F, Jeanmougin F, Higgins DG (1997). The CLUSTAL_X windows interface: flexible strategies for multiple sequence alignment aided by quality analysis tools. Nucleic Acids Res.

[B52] de Oliveira T, Deforche K, Cassol S, Salminen M, Paraskevis D, Seebregts C, Snoeck J, van Rensburg EJ, Wensing AM, Vijver DA van de, Boucher CA, Camacho R, Vandamme AM (2005). An automated genotyping system for analysis of HIV-1 and other microbial sequences. Bioinformatics.

[B53] Ray S Simplot v2.5.0. http://sray.med.som.jhmi.edu/SCRoftware/simplot/.

[B54] Posada D, Crandall KA (1998). MODELTEST: testing the model of DNA substitution. Bioinformatics.

[B55] Ronquist F, Huelsenbeck JP (2003). MrBayes 3: Bayesian phylogenetic inference under mixed models. Bioinformatics.

[B56] Rambaut A, Drummond A (2007). Tracer v1.4. http://beast.bio.ed.ac.uk/Tracer.

[B57] Guindon S, Gascuel O (2003). A simple, fast, and accurate algorithm to estimate large phylogenies by maximum likelihood. Syst Biol.

[B58] Guindon S, Lethiec F, Duroux P, Gascuel O (2005). PHYML Online – a web server for fast maximum likelihood-based phylogenetic inference. Nucleic Acids Res.

[B59] Drummond AJ, Nicholls GK, Rodrigo AG, Solomon W (2002). Estimating mutation parameters, population history and genealogy simultaneously from temporally spaced sequence data. Genetics.

[B60] Drummond AJ, Rambaut A (2007). BEAST: Bayesian evolutionary analysis by sampling trees. BMC Evol Biol.

[B61] Drummond AJ, Rambaut A, Shapiro B, Pybus OG (2005). Bayesian coalescent inference of past population dynamics from molecular sequences. Mol Biol Evol.

[B62] Drummond AJ, Ho SY, Phillips MJ, Rambaut A (2006). Relaxed phylogenetics and dating with confidence. PLoS Biol.

